# Breaking Down Glioma-Microenvironment Crosstalk

**DOI:** 10.1177/10738584241259773

**Published:** 2024-07-26

**Authors:** Raghavskandhan Ramachandran, Alexander F. Jeans

**Affiliations:** 1 Balliol College, University of Oxford, Oxford, UK; 2 Department of Pharmacology, University of Oxford, Oxford, UK

**Keywords:** glioma, glioblastoma, neurologic disease, neurooncology, synapse, host-glioma interactions, high-grade glioma

## Abstract

High-grade gliomas (HGGs) are the commonest primary brain cancers. They are characterized by a pattern of aggressive growth and diffuse infiltration of the host brain that severely limits the efficacy of conventional treatments and patient outcomes, which remain generally poor. Recent work has described a suite of mechanisms via which HGGs interact, predominantly bidirectionally, with various cell types in the host brain including neurons, glial cells, immune cells, and vascular elements to drive tumor growth and invasion. These insights have the potential to inspire novel approaches to HGG therapy that are critically needed. This review explores HGG–host brain interactions and considers whether and how they might be exploited for therapeutic gain.

## Introduction

Most primary brain tumors are gliomas, which are derived from glial cells or their precursors. The gliomas are a diverse group of entities that range from essentially benign to highly malignant, with profoundly different genetic rearrangements underlying the different subtypes. In this review, we focus on the more aggressive (i.e., high histopathologic grade) gliomas of the “diffuse” lineage, a designation that refers to their typical growth habit. This group includes diffuse astrocytoma, oligodendroglioma, and glioblastoma, entities that have recently been even further resolved into a number of distinct, largely molecularly defined diagnostic categories (Louis and others 2021). Nonetheless, they can be collected under the umbrella term *high-grade glioma* (HGG) and share many commonalities in terms of genetics and behavior.

**Box 1.** Glioma

Gliomas are neoplasms that originate from glial cells in the central nervous system. They span the entire spectrum of malignancy—from extremely slow-growing and entirely benign entities all the way up to some of the most aggressive human malignancies known. As with most brain tumors, the symptoms of gliomas can arise from any combination of raised intracranial pressure, the epileptogenic nature of space-occupying lesions within the brain, and local damage to brain regions with specific functions neighboring the tumor. Therefore, headaches, vomiting, seizures (focal or generalized), and focal neurologic signs are all possible presentations.Gliomas are broadly classified according to their cellular (microscopic) morphology, specifically the line of differentiation that this suggests—for example, astrocytoma (astrocytic differentiation), oligodendroglioma (oligodendroglial differentiation), ependymoma (ependymal), and so on. Each glioma is also classified by its grade on a scale of 1 to 4 devised by the World Health Organization (WHO), and this WHO grade is an index of the number of cellular features that are associated with aggressive behavior that the tumor shows on microscopic examination, such as mitotic activity, neovascularization, or necrosis. Grade 1 tumors show a benign course and are normally dependent on anatomic location and cured by resection alone. Grade 4 tumors are highly aggressive malignancies that are treated with surgery alongside adjunct therapies such as radiotherapy and chemotherapy. Unfortunately, many of these still carry a dismal prognosis.The classification of gliomas has undergone a further overhaul in recent years as the presence or absence of specific gene mutations that are known to drive tumor growth (driver mutations) is taken into account. Some of these mutations are now considered entity defining; for instance, to be classified as an oligodendroglioma, a tumor must not only have the appropriate microscopic appearance, but also harbor both a mutation in isocitrate dehydrogenase and a combined 1p/19q chromosomal deletion.

HGGs are aggressive, often lethal neoplasms that interact intimately with the surrounding host brain, sharing a tendency to infiltrate and invade widely. Seminal histologic characterization conducted by Scherer (1938) in the 1930s began to define these interactions morphologically; however, over recent decades the focus of such studies has naturally changed, and research has begun to probe the genetic and molecular underpinnings of host-glioma interactions, unveiling significant complexity. Molecular signals from multiple cell types in the glioma microenvironment, including astrocytes and neurons, have been shown to promote HGG growth and invasion (Kim and others 2014; Venkatesh and others 2015; Zhang and others 2012). However, this is only a part of the story, as the tumor cells in turn appear to play an active role in the modulation of surrounding host cells, promoting the formation of a microenvironment conducive to tumor growth and proliferation (Tamai and others 2022). This bidirectional communication between HGG and the host brain constitutes a critical foundation for their invasive behavior and progression.

Long-term survival rates following an HGG diagnosis remain low (Schaff and Mellinghoff 2023). Surgical debulking is currently the first-line treatment of choice, but recurrence rates are extremely high because of the diffuse growth pattern and widely invasive nature of these tumors, making complete resection effectively impossible (Sahm and others 2012). In addition, the vulnerability of the CNS and the intrinsic resilience of HGG limit the effectiveness of chemotherapy and radiotherapy (Weil and others 2017). There is therefore an acute need for new and ideally targeted treatment options. Significant recent progress in understanding host-glioma interactions builds on an existing body of work in this area (Watkins and Sontheimer 2012) and raises the hope of identifying novel mechanisms underlying tumor growth and survival that could potentially be leveraged for therapeutic gain. This review aims to outline the key recent findings, drawing out those that are most pathophysiologically significant to consider whether they might provide a basis for the novel therapies that will be so critical if the prognosis of these clinically highly challenging tumors is to be transformed.

**Box 2.** Current Treatment for Glioma

Treatment strategies for glioma are variable and depend on the classification of the tumor. The most benign lesions (WHO grade 1) can usually be cured by complete resection, assuming that their location within the brain allows this. Treatment of higher-grade lesions may also involve various combinations of radiotherapy, standard chemotherapy, and, increasingly, agents targeted at proteins carrying specific driver mutations. Definitive diagnosis and classification of gliomas require histologic examination of a tissue biopsy, but preoperative MRI provides an increasingly detailed and accurate account of the structure and anatomy of the lesion, which in many cases is sufficient for diagnosis with a high degree of confidence.The commonest high-grade glioma in adult patients, glioblastoma, is unfortunately one of the most malignant. This WHO grade 4 tumor is usually treated aggressively with surgery, radiotherapy, and chemotherapy, but outcomes remain poor, with median survival with treatment being only 1 to 2 years depending on the age group. One of the greatest therapeutic challenges is the diffusely infiltrative nature of the tumor, with complete surgical resection being effectively impossible. However, the characterization of molecular changes in these tumors is constantly improving, and some of these changes have therapeutic significance. For instance, glioblastomas with an inactivating hypermethylation of the promoter of the MGMT gene (O6-methylguanine-DNA methyltransferase) exhibit defective DNA repair in response to alkylation and are therefore sensitive to alkylating chemotherapy agents such as temozolomide. As such, glioblastomas usually undergo testing for MGMT promoter hypermethylation, and the results are used, with other relevant patient data, to stratify cases into those receiving temozolomide or alternative treatments. In some US centers, a fourth treatment modality has recently been added for which there is some evidence of effectiveness: tumor-treating fields, in which growth-inhibiting alternating electric fields are administered directly to the patient’s scalp (see main text).

## Tumor Progression and Neuronal Activity Are Intertwined

**Figure 1.**
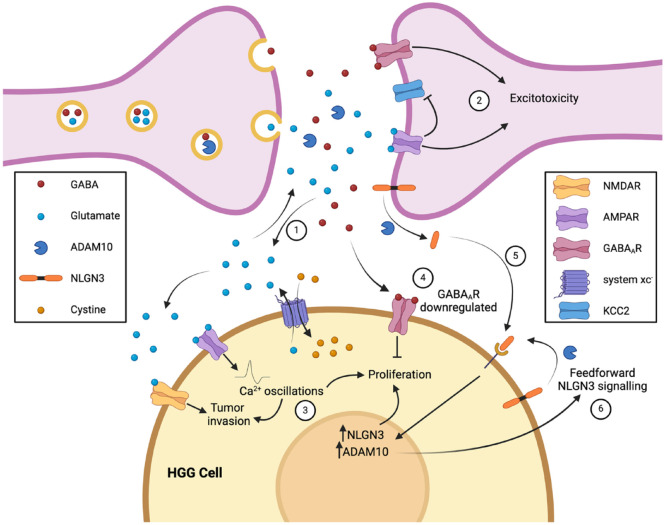
High-grade gliomas (HGGs) interact with neurons through a variety of signals, including neurotransmitters (glutamate, GABA) and neurotrophic factors (NLGN3). Synaptic glutamate release is complemented by glutamate release by HGG cells through system xc^–^, a cystine-glutamate antiporter (1). The excess glutamate leads to excitotoxicity in neurons, potentially generating space for glioma growth (2). HGG cells themselves express AMPA and NMDA receptors, although in lower numbers to protect them from excitotoxicity; instead, paracrine and autocrine glutamate signaling promotes glioma invasion and proliferation (3). In contrast, paracrine GABA signaling inhibits HGG proliferation, leading to glioma cells down-regulating GABA_A_ receptors as they transform from low- to high-grade tumors to mitigate this effect (4). NLGN3, a synaptic adhesion protein, is another mediator of glioma proliferation. NLGN3 is shed from the synaptic membrane when it is cleaved by ADAM10, an enzyme released in an activity-dependent manner from synaptic vesicles, enabling NLGN3 to bind to HGG cells and promote proliferation (5). The shed NLGN3 also generates a feedforward signaling loop by increasing expression of ADAM10 and NLGN3 within glioma cells (6). Created with Biorender.com.

HGG cells have been known to associate closely with neurons at least since Scherer’s (1938) influential histologic work, which demonstrated clear perineuronal patterns of tumor growth, with cuffs of cells surrounding and directly contacting neurons. Recent work has begun to explore some of the molecular interactions that mediate or are facilitated by glioma-neuron associations, one of which is paracrine signaling via neurotransmitters (Figure 1). Gliomas respond to a variety of neurotransmitters, including glutamate (Labrakakis and others 1998b; Lyons and others 2007) and GABA (Labrakakis and others 1998a). There is some evidence that functional GABA_A_ receptors appear to be present on low-grade gliomas but not on HGG, implicating a loss of GABA_A_ receptors in the transformation to a high-grade phenotype (Labrakakis and others 1998a). Indeed, a potential functional role for GABA_A_ receptor loss in driving tumor growth is suggested by the ability of GABAergic afferent activity to limit the physiologic proliferation of oligodendrocyte precursor cells (OPCs; Liu and others 2011); this is significant because OPCs, with neural stem cells and astrocytes, are prime candidates for the cell of origin of gliomas (Zong and others 2015). However, the existence of this mechanism remains speculative, and further functional studies are required to confirm a definite role for GABA signaling in glioma growth and progression.

The evidence for a role for glutamate in driving tumor growth and progression, however, is much clearer. Glutamate regulates glioma growth in a number of ways. HGG cells release glutamate in the process of cystine uptake with system xc^–^, a cystine-glutamate exchanger (Lyons and others 2007), and this glioma-derived glutamate activates Ca^2+^-permeable α-amino-3-hydroxy-5-methyl-4-isoxazolepropionic acid receptors (AMPARs) expressed on the same or neighboring cells in an autocrine or paracrine manner (Figure 1). AMPAR activation induces intracellular Ca^2+^ oscillations that are essential for cell migration in HGG (Lyons and others 2007), and similar Ca^2+^ oscillations have been implicated in driving proliferation (Hausmann and others 2022). *N*-methyl-d-aspartate receptors (NMDARs), the other principal glutamate receptor type, may play a role in HGG growth, although they seem to be less important than AMPAR. While some earlier studies suggested that NMDARs were not even expressed on glioma cells, later work indicated that NMDAR stimulation does in fact enhance tumor invasiveness while NMDAR blockade prevents this effect (So and others 2021).

Glutamate release by HGG leads to an elevation of extracellular glutamate concentration that can drive excitotoxic death in surrounding neurons. This manifests as tumor necrosis, a histopathologic signature of HGG that is one of the diagnostic criteria for these tumors. Although necrosis may be, to some degree, a simple consequence of the rapid growth of HGG outstripping vascular supply (Raza and others 2002), it has been proposed that it may actually be beneficial for tumor growth by creating space for neoplastic cells to occupy within the highly constrained environment of the brain and skull (Sontheimer 2008). Elevation of extracellular glutamate levels is exacerbated by enhanced activity of peritumoral neuronal synapses, which is associated with the development of seizures (Campbell and others 2012). This excess synaptic activity is driven in part by glioma-derived glutamate, which can cause down-regulation in surrounding neurons of the potassium chloride cotransporter (KCC2) that normally extrudes Cl^–^ to maintain the GABA reversal potential (Campbell and others 2015); lowering KCC2 expression increases intracellular Cl^–^, in turn leading to a shift in neuronal GABA_A_ receptor signaling from hyperpolarizing to depolarizing and thereby promoting peritumoral neuronal hyperexcitability (MacKenzie and others 2016). The tumor cells themselves can limit the excitotoxic effects of excess extracellular glutamate by attenuating their AMPAR expression (van Vuurden and others 2009). Through these mechanisms, glutamate release by tumor cells and neighboring neurons generates a positive feedback cycle driving further glutamate release via increased synaptic activity and promoting tumor growth and progression partly facilitated by the degradation and necrosis of surrounding nontumoral host brain.

Besides neurotransmitters, neuron-glioma interactions can involve a variety of other secreted neuronal activity–regulated molecules. An initial observation that neuronal activity exerts a mitogenic effect on normal nontumoral OPCs (Gibson and others 2014) led to a hypothesis that it might also promote proliferation in HGG, for which OPCs are one of the leading candidates for the cell of origin. This was tested by optogenetic control of neuronal activity in a patient-derived HGG xenograft model, which confirmed that neuronal activity does indeed promote glioma growth (Venkatesh and others 2015). A subsequent search for activity-regulated secreted factors potentially mediating this effect revealed that, in addition to known tumor mitogens such as BDNF, the synaptic adhesion molecule neuroligin 3 (NLGN3), previously unknown in this context, exerted a particularly robust effect (Venkatesh and others 2015). In a follow-up study, the authors used immunodeficient NLGN3 knockout mice bearing xenografts of patient-derived HGG cells to show that, in the absence of NLGN3, tumor growth was almost completely inhibited over a protracted observation period of 6 months (Venkatesh and others 2017). This study also identified ADAM10, released from neurons in an activity-dependent manner, as the enzyme responsible for the cleavage and shedding of NLGN3, and it delineated a number of downstream signaling pathways through which NLGN3 exerts its mitogenic effects (Venkatesh and others 2017). Moreover, it appears that NLGN3 may initiate a feedforward loop by up-regulating ADAM10 to increase its own production and secretion (Dang and others 2021).

**Figure 2.**
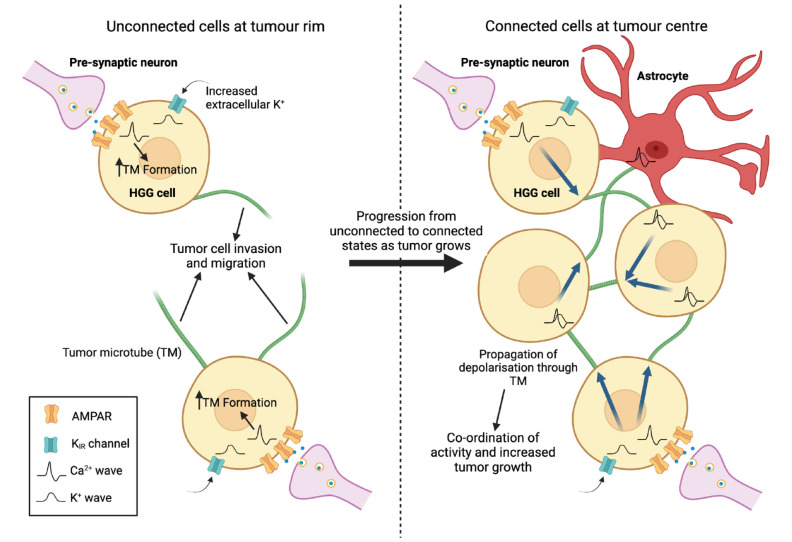
Neurons form functional synapses with a small proportion of high-grade glioma (HGG) cells in the tumor mass. At the tumor rim (left panel), synapses form between neurons and individual tumor cells unconnected to the existing glioma-astrocyte network in the tumor mass. Ca^2+^ oscillations generated by AMPA receptor activation drive tumor microtube (TM) formation, which enhances the ability of tumor cells to migrate and invade. Over time, TMs anchor to other tumor cells or astrocytes, and the connected cells become part of the glioma-astrocyte network that constitutes the main tumor mass (right panel). As connected tumor cells undergo a change in their gene expression profiles, the Ca^2+^ waves produced at neuron-glioma synapses and the nonsynaptic depolarizing inward K^+^ currents that result from accumulation of K^+^ due to elevated synaptic activity can propagate through TMs to the rest of the network, synchronizing the activity of tumor cells and promoting growth. The glioma-astrocyte network further functions to protect the tumor and enable recovery from injury, including therapeutic methods such as radiotherapy. Created with Biorender.com.

Together, these findings established a complex and bidirectional relationship between neuronal activity in the host brain and tumor growth (Watkins and Sontheimer 2012), which not only represented a novel mechanistic paradigm in neurooncology but also identified a number of novel potential molecular targets for antiglioma therapeutics. However, an even more direct link would emerge in a series of studies published over the last 4 years that extended the concept of active bidirectional regulation even further. Building on the observation that functional synapses exist between neurons and OPCs (Bergles and others 2000), studies from two independent groups demonstrated the formation of functional neuron-glioma synapses (Figure 2). Both studies in question provided clear evidence at the level of ultrastructure (electron microscopy) and function (electrophysiology) that host neurons form glutamatergic chemical synapses between presynaptic host neurons and postsynaptic HGG cells (Venkataramani and others 2019; Venkatesh and others 2019). These synapses generate postsynaptic AMPAR-mediated currents that initiate intracellular signaling driving tumor growth and host brain invasion; furthermore, pharmacologic blockade of these synapses inhibits growth and extends survival of mouse HGG xenograft models. While it appears that only 5% to 10% of tumor cells receive direct synaptic input from neurons (Venkatesh and others 2019), at least some of these cells may represent a subpopulation with a particularly invasive phenotype that is regulated by neuronal activity (Venkataramani and others 2022). Such cells fuel the extensive colonization of the brain that is a feature of HGG and is one of the major challenges in treatment. Yet, over time and under the continued influence of neuronal input resulting in intracellular Ca^2+^ transients, they gradually transition into a more stable population that is highly interconnected via tumor microtubules (TMs) and has less propensity to migrate (Venkataramani and others 2022). TMs are protrusions of the cell membrane that couple glioma cells through gap junctions, and the TM network is important for a number of reasons. TM-connected cells are relatively radiotherapy resistant, an effect that appears to be mediated through the homeostasis or buffering of damage-induced increases in Ca^2+^ levels in individual cells that the integrated TM-connected network allows (Osswald and others 2015). In addition, the TM network propagates and amplifies both Ca^2+^ transients and a nonsynaptic K^+^-mediated prolonged inward depolarization that results from extracellular K^+^ accumulation due to synaptic activity, both of which drive HGG proliferation (Venkatesh and others 2019). In keeping with these observations, blockade of synaptic activity and gap junctions inhibits growth and extends survival in mouse xenograft models (Venkataramani and others 2019; Venkatesh and others 2019).

While the work detailed so far focuses predominantly on interactions at the levels of cells and synapses, there are neuronal network-level consequences of these phenomena. By using electrical recordings and biopsy material taken from patients undergoing awake surgery for HGG resection, it was found that the presence of tumor functionally remodels circuits such that the neural response to a task in a tumor-infiltrated brain involves the activation of a much larger region than would be recruited in a healthy brain (Krishna and others 2023). This remodeling, which appears to be at least partly dependent on the known astrocyte-secreted synaptogenic factor thrombospondin 1, promotes tumor proliferation and progression and impairs cognition. Gabapentin, which blocks the thrombospondin receptor α2δ-1, resulted in a marked decrease in tumor cell proliferation in mice bearing HGG xenografts (Krishna and others 2023). If increased neural activity at the synaptic or network level in the brain acts as a driver of growth in HGG, the question arises whether external inputs that ordinarily bring about such increases in activity, for instance sensory experience, can initiate or drive glioma growth. Indeed, it has been shown in a genetic mouse model of HGG that olfactory experience associated with increased activity in olfactory neurons promotes gliomagenesis in the olfactory bulb via neuronal insulin-like growth factor 1 release (P Chen and others 2022). This mechanism also appears to be relevant in a pathologic setting: the initiation of optic pathway gliomas in a mouse model of the human cancer predisposition syndrome neurofibromatosis 1 has been shown to depend on optic nerve activity and consequent NLGN3 secretion driven by visual experience (Pan and others 2021). Depriving the animals of visual experience during a critical tumorigenic developmental window prevents tumor formation and maintenance.

A relatively short period has seen a wealth of evidence accumulating in support of a complex model of indirect and direct coregulatory interactions of host neurons and neoplastic cells that is not only revolutionizing our view of the biology of HGG but perhaps, most important, identifying a number of potential therapeutic targets. While most of these are molecular in nature, there is the remarkable possibility that manipulation of host sensory experience might inhibit tumor growth via modulation of neural activity in relevant cortical and subcortical areas. Excitement surrounding this apparent profusion of novel molecules and processes with possible therapeutic potential must be tempered by the realization that most of those described here are, because of their very recent discovery, at an extremely early stage in the transition from bench to bedside. Experimental treatments based on these candidates are just being designed and, to the best of our knowledge, have yet to reach clinical trials. Statistically, most will fail at some stage of this lengthy pathway to translation. Nonetheless, the promise, even distant, of any novel and effective approach in this area of unmet need must be regarded as genuine cause for optimism.

## Reactive Astrocytes Interact With HGGs to Drive Growth and Proliferation

**Figure 3.**
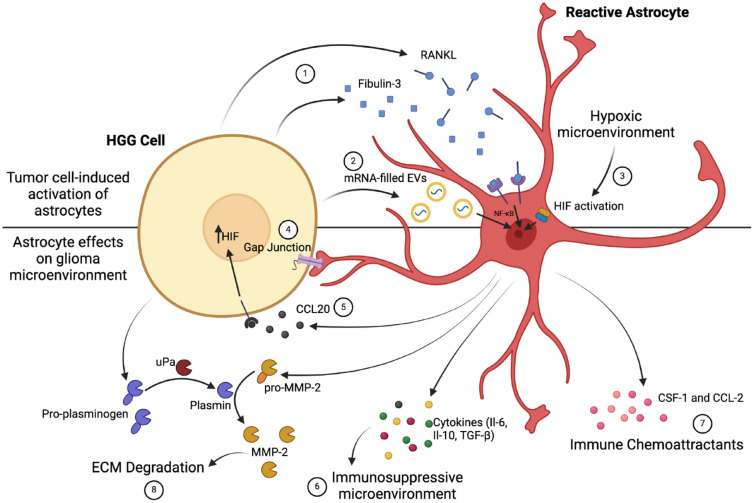
Astrocytes can be activated by high-grade glioma (HGG) to form protumor reactive astrocytes (top). HGGs release multiple factors that can lead to this transformation of astrocytes, such as fibulin 3 and RANK-L, which act through the NF-κB pathway (1). HGGs have also been shown to produce extracellular vesicles (EVs) containing mRNAs, which can lead to the transcription changes within astrocytes that enable a reactive phenotype (2). Furthermore, the activity of HGG results in local hypoxia, which can activate the hypoxic-inducible factor (HIF) pathway and indirectly produce reactive astrocytes (3). The resulting reactive astrocytes shape the microenvironment and promote tumor invasiveness (bottom). Reactive astrocytes form gap junctions with HGG cells to facilitate direct communication and regulate growth (4). Reactive astrocytes also produce CCL20, a cytokine that acts on tumor cells to up-regulate HIF production, enhancing the activity of HIF-1–dependent pathways that facilitate growth (5). Reactive astrocytes release immunosuppressive cytokines, further regulating the microenvironment (6), as well as recruiting immune cells that are reprogrammed into a tumor-supportive phenotype (7). Finally, they degrade the extracellular matrix (ECM) to allow tumor growth (8). Created with Biorender.com.

Astrocytes are cells that play a key role in supporting neuronal function in the CNS, and they have central roles in many processes, including ion and neurotransmitter homeostasis, regulation of cerebral blood flow, and repair of neural tissue. Following direct brain injury or a variety of other insults, such as ischemia, quiescent astrocytes can undergo a set of morphologic, transcriptomic, and functional changes to become “reactive,” which enables them to fulfill their critical roles in these situations. Reactive astrocytes are not a single cell type; instead, the term encompasses a spectrum of related changes, which means that they may constitute a heterogeneous population even at a single site (Escartin and others 2021). Reactive change can also be induced by a variety of disease states, including glioma (Figure 3, top). While morphologic evidence of this phenomenon has long been noted by neuropathologists during diagnostic histologic examination of tumors, it has been more formally and comprehensively demonstrated in experimental systems such as human stem cell–derived astrocyte and HGG cell cocultures (Gagliano and others 2009). The mechanisms through which HGG cells can elicit reactive change in astrocytes are not entirely clear, but there is evidence that they can produce RANK-L and fibulin 3, which activate astrocytes through the NF-κB pathway (Kim and others 2014). More recent work has also implicated a role for the hypoxic-inducible factor (HIF) pathway, which can be activated in the areas surrounding the necrotic regions that are characteristic of HGG (Pantazopoulou and others 2021). The transfer of mRNAs packaged in extracellular vesicles from glioma cells constitutes a further candidate mechanism for the generation of reactive astrocytes (Gao and others 2020; Zeng and others 2020).

These observations raise the question, of course, of whether and how reactive astrocytes might contribute to HGG growth and progression, and indeed there are a number of mechanisms through which this occurs. Reactive astrocytes show increased expression of active and inactive precursor forms of matrix metalloproteinase 2 (MMP-2), an enzyme that is able to degrade the extracellular matrix, directly enabling tumor invasion (Figure 3, bottom). HGG can harness the inactive MMP-2 through the production of plasminogen, which is then cleaved by urokinase-type plasminogen activator to plasmin. Plasmin in turn cleaves pro–MMP-2 to its active form (Le and others 2003). Reactive astrocytes can further contribute to HGG progression through the release of various cytokines, including IL-6 (Chen and others 2016), IL-10 (Henrik Heiland and others 2019), and TGF-β (Kim and others 2014), that promote the formation of an immunosuppressive environment, blunting the immune response to tumor cells. The expression of CCL-2 and CSF-1 by reactive astrocytes is particularly significant, as it appears to regulate the recruitment of tumor-associated macrophages and their subsequent reprogramming into a phenotype that supports HGG growth and invasion (Perelroizen and others 2022). Astrocytes can also potentiate the response of HGG to hypoxia through the release of CCL20, which up-regulates HIF-1, enhancing the activity of HIF-1–dependent pathways that facilitate growth under hypoxic conditions (Jin and others 2018).

Reactive astrocytes can directly communicate with HGG through gap junctions involving connexin 43 (Cx43), a protein that is normally up-regulated in reactive astrocytes (Zhang and others 1999). There is a body of evidence indicating that Cx43 acts as a tumor suppressor in HGG (Jaraiz-Rodriguez and others 2020). Cx43 exerts this effect through several mechanisms, including altering the expression of key cell cycle proteins so that the transition to S and M phases is inhibited (Sanchez-Alvarez and others 2006). Moreover, in order to inhibit tumor growth, Cx43 may modify the expression of other growth regulators, such as IGF-1 (Bradshaw and others 1993), and a number of genes associated with the maintenance of stemness (Jaraiz-Rodriguez and others 2020), a critical property in sustaining the growth and progression of neoplastic lineages. Despite this, the role of Cx43 is not straightforward, as there is evidence that this protein can facilitate glioma cell migration and invasion (Sin and others 2016). The mechanism through which this occurs is not yet clear. Some studies suggest that it may be independent of the channel function of Cx43 given that, unlike a simple Cx43 knockdown, overexpression of a channel-defective but otherwise intact Cx43 in glioma cells has been found to have no effect on invasion (Sin and others 2016). This conclusion is supported by previous work from the same laboratory showing that the cytoplasmic tail of Cx43 was sufficient to promote motility in HGG cells in vitro (Crespin and others 2010), and that study demonstrated that the effect was independent of gap junction intercellular communication as assessed by dye transfer. However, these results appear at odds with the findings of earlier studies, which found that Cx43 promotes migration in a channel-dependent manner (Lin and others 2002; Oliveira and others 2005). It may be, therefore, that Cx43 regulates the invasiveness of HGG via multiple mechanisms. More recent work has suggested that microRNAs can be transferred in a Cx43-dependent but channel-independent manner from glioma cells to astrocytes to weaken astrocyte adhesion to the basement membrane, facilitating HGG invasion (McCutcheon and Spray 2022). These authors also showed that Cx43 may regulate invasiveness in opposite directions when expressed in neoplastic cells versus in astrocytes, adding further complexity to this already challenging question but suggesting a potential explanation for some of the previous conflicting results. While it is clear overall that Cx43-mediated contact with astrocytes is a key regulator of growth and invasiveness in HGG, it seems that greater understanding of the mechanistic details underlying these roles will be required to unlock the potential therapeutic applications of this interaction.

## Immunosuppressive Tumor Microenvironment Supports HGG Growth

**Figure 4.**
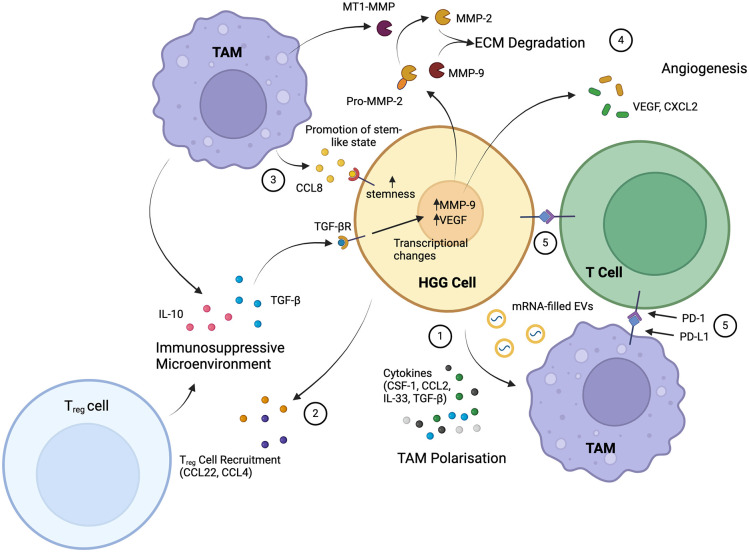
The key interactions of high-grade glioma (HGG) and immune cells involve tumor-associated macrophages (TAMs) and T lymphocytes. HGGs induce M2 polarization of TAMs, making them immunosuppressive and tumor supportive, through the release of cytokines and mRNA-filled extracellular vesicles (EVs) (1). The release of other cytokines, as well as the hypoxic environment, recruits regulatory T (T_reg_) cells, another immunosuppressive cell (2). Recruited TAMs reciprocate HGG signaling with further release of immunosuppressive cytokines (IL-10 and TGF-β), as well as the release of CCL8, which promotes glioma stemness (3). TGF-β induces transcriptional changes in tumor cells, which function to induce degradation of the extracellular matrix (ECM) by metalloproteinases and stimulate angiogenesis in the microenvironment (4). A further key immunosuppressive mechanism is the direct interaction between tumor cells and TAMs with T lymphocytes through PD-L1 to inhibit T-lymphocyte function (5). Overall, HGGs establish a strongly immunosuppressive microenvironment with tumor-supportive cells that enables growth and progression. Created with Biorender.com.

The HGG microenvironment is heavily infiltrated and surveyed by immune cells (Buonfiglioli and Hambardzumyan 2021), and tumor cells must therefore employ a variety of immunosuppressive mechanisms to prevent their own destruction. These mechanisms depend on bidirectional interactions between neoplastic and immune cells, many of which not only suppress the antitumor immune response but also facilitate processes that actively drive tumor growth (Figure 4). HGG can recruit a variety of immune cells, including regulatory T lymphocytes, microglia, myeloid-derived suppressor cells, and peripheral macrophages (Gieryng and others 2017), and this is accomplished through the production of chemokines such as CCL-2 (Zhang and others 2012), CSF-1, and EGF (Coniglio and others 2012). The majority of immune cells associated with HGG are of the macrophage/microglial lineage, termed *tumor-associated macrophages/microglia* (TAMs). The gene expression profile of recruited TAMs becomes distinctly immunosuppressive, protecting tumor cells from immune destruction (Gieryng and others 2017), although the mechanisms through which this immunosuppressive phenotype is induced are not yet entirely clear. There is some evidence that the release of cytokines, including TGF-β (Buonfiglioli and Hambardzumyan 2021), CSF-1 (Pyonteck and others 2013), CCL-2 (Zhang and others 2012), and IL-33 (De Boeck and others 2020), by tumor cells can polarize TAMs toward what is termed the *M2 phenotype*, an immunosuppressive state that contrasts with the more usual proinflammatory M1 phenotype. In addition, HGG cells appear able to release microRNAs packaged in extracellular vesicles that are taken up avidly by microglia and have the effect of shifting them toward immunosuppression (van der Vos and others 2016). It is, however, not only the tumor cells themselves that drive immunosuppression in the tumor microenvironment, given that TAMs can be reprogrammed through interactions with reactive astrocytes as mentioned previously (Perelroizen and others 2022). Macrophages and microglia in turn release effectors that can promote the generation of reactive astrocytes, potentially leading to a positive feedback loop with greatly elevated local concentrations of anti-inflammatory cytokines (Henrik Heiland and others 2019).

In addition to driving local immunosuppression, TAMs exhibit tumor-supportive phenotypes that promote the growth and proliferation of HGG through a variety of additional mechanisms (Buonfiglioli and Hambardzumyan 2021; Zhai and others 2011). TAM-secreted TGF-β, for example, has a number of additional effects, such as the promotion of HGG invasiveness and the direct stimulation of HGG to produce MMP-9 (Ye and others 2012) and pro–MMP-2. Pro–MMP-2 is activated when it is cleaved by macrophage-produced membrane type 1 metalloproteinase, the production of which is induced by glioma cells through TLR2 signaling (Markovic and others 2009). MMP-9 and MMP-2 together contribute to the destruction of the extracellular matrix and progressive tumor invasion. TAM-derived TGF-β induces the release of angiogenic factors from tumor cells, such as vascular endothelial growth factor (VEGF; Koochekpour and others 1996). Other angiogenic factors are released by TAMs themselves, including the chemokine CXCL2 (Brandenburg and others 2016), and together these drive the growth of new blood vessels that support the metabolic demands associated with HGG growth. VEGF and CXCL2 also have immunosuppressive effects, underlining the pleiotropic roles of many cytokines in this context (Wang and others 2021). A further protumoral role of TAMs is the maintenance of stemness of glioma stem cells through CCL8 signaling (Zhang and others 2020). CCL8 binds to the receptors CCR1 and CCR5 and activates Erk1/2 downstream to stimulate stemness and, simultaneously, invasiveness through the growth of pseudopodia.

Immune cells other than microglia/macrophages can also exert significant effects on HGG growth. Dendritic cells are antigen-presenting cells that interact directly with T lymphocytes and can activate signaling pathways to promote the recognition and destruction of tumor cells (Friedrich and others 2022). However, HGG employ multiple mechanisms to subvert this process. In the tumor microenvironment, dendritic cells can be induced by HGG cells to overexpress Nrf2, blocking their maturation and decreasing their ability to activate effector T cells (Wang and others 2017). Antitumor effects of T cells are also inhibited by several cytokines released by TAMs and tumor cells themselves (Ravi and others 2022). Another mechanism by which HGGs evade the T-cell response is the expression of PD-L1, which binds to PD-1 on T cells and suppresses their proliferation and immune function (Goods and others 2017; Zeng and others 2013). HGG can also induce the expression of PD-L1 by TAMs (Bloch and others 2013).

A significant proportion of T cells are regulatory T cells (T_regs_), an important subpopulation with a critical role in regulating and suppressing the activity of other immune cells. T_regs_ can be attracted by the HGG-secreted chemokine CCL2, and blockade of this chemokine’s receptor—or that of the receptor for another chemokine, CCL22, which is employed by several tumor types to attract T_regs_—prevents T_reg_ migration, indicating that multiple signaling pathways can mediate this important role (Jordan and others 2008). In addition to attracting migratory T_regs_, HGG can induce the T_reg_ phenotype in naïve T cells, an effect that is potentiated in the hypoxic tumor microenvironment through HIF-1α signaling (Wei and others 2011). HGGs accumulate another immune cell type that can suppress the T-cell response: the relatively newly described myeloid-derived suppressor cell (Kamran and others 2017). The overall view that emerges from these studies is that HGGs are able recruit a host of mechanisms to subvert and avoid the effects of the powerful host antitumor immune response. As with the other host-glioma interactions that we have discussed, this field of study is still relatively young; nonetheless, as these largely immune suppressive interactions clearly play a key role in tumor cell survival, understanding them as thoroughly as possible will surely reveal novel approaches to effective therapy. Indeed, to some extent this has already happened, as described later in this article, although these approaches remain at an early stage of development.

## Crosstalk Between HGG and Endothelial Cells Underpins Angiogenesis

Neoangiogenesis, usually resulting in extensive tumor vasculature, is a hallmark of HGG (Buccarelli and others 2022). Angiogenesis is a physiologic process that takes place throughout life, as needed for the remodeling of local vascular networks during growth, or in response to injury. One trigger for this process is tissue hypoxia, which leads to the induction of the protein HIF-1α and downstream release of angiogenic factors such as VEGF. HGGs can exploit these mechanisms to enhance their own vascular supply, thereby enabling growth. This can occur through direct release of angiogenic factors by tumor cells or indirectly by the release of exosome-packaged long noncoding RNAs that induce the expression of angiogenic factors in the endothelial cells themselves (Lang and others 2017; Treps and others 2017). Similarly, HGG cells can transfer another noncoding RNA type, microRNAs, to endothelial cells via gap junctions to promote angiogenesis (Thuringer and others 2016). Aside from the induction or manipulation of secreted angiogenic factors leading to classical angiogenesis, at least three separate additional mechanisms leading to tumor neovascularization have been identified in HGG: vascular co-option, vascular mimicry, and glioma cell transdifferentiation (Buccarelli and others 2022).

**Figure 5.**
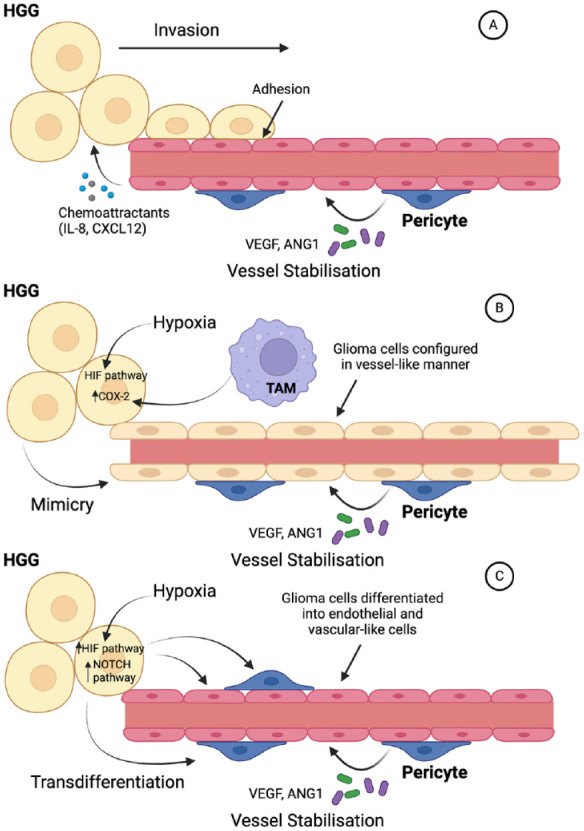
Angiogenic mechanisms utilized by high-grade glioma (HGG). (A) Vascular co-option in which tumor cells are recruited by chemoattractants, including IL-8 and CXCL12, and adhere to endothelial cells, leading to invasion along the course of a blood vessel. This enables the incorporation of existing vessels into the tumor bulk. As this mechanism does not involve the generation of new blood vessels, it is often essential early in glioma development. (B) Vascular mimicry in which tumor cells themselves form vessel-like structures. Hypoxia is a major stimulus for vascular mimicry, with tumor-associated macrophages (TAMs) being an important secondary factor. (C) Glioma transdifferentiation portrays HGG cells converting to endothelium and pericytes to generate and support new vessels. The NOTCH pathway is critical for stimulating glioma transdifferentiation. In all scenarios, the presence of pericytes is key to stabilizing vessels so that they are able to support tumor growth. Created with Biorender.com. HIF = hypoxic-inducible factor.

Vascular co-option is the recruitment, or co-option, of existing blood vessels into the tumor mass (Figure 5A), a process that is independent of VEGF. Endothelial cells are critical for this process because they release IL-8 and CXCL12, which stimulate HGG invasion and act as chemokines attracting tumor cells toward blood vessels (McCoy and others 2019; Yadav and others 2016). Subsequently, direct adhesion molecule–mediated interactions between HGG and endothelial cells anchor the tumor to the blood vessels (Burgett and others 2016). Other cells within the complex tumor microenvironment support the process, such as astrocytes and immune cells that release matrix metalloproteinases, breaking down extracellular matrix to facilitate tumor invasion. One consequence of vascular co-option is that vessels encompassed by the tumor bulk can become compressed, leading to hypoxia and the release of VEGF, stimulating additional true neovascularization (Voutouri and others 2019).

Hypoxia is a major driving factor for the two highly related processes of vascular mimicry and glioma cell transdifferentiation. Vascular mimicry involves the formation of vessel-like structures by tumor cells that lack endothelium (Figure 5b), while in cell transdifferentiation, pluripotent tumor stem cells differentiate toward an endothelial cell phenotype and form new vessels (Figure 5c). In both processes, tissue hypoxia drives the expression of the required signaling molecules and effectors, such as adhesion molecules, largely via HIF-1α (Mao and others 2013; Rocha and others 2018). VEGF (Yao and others 2013) and various noncoding RNAs (Xue and others 2016) are also implicated in vascular mimicry, as are tumor-associated macrophages via cyclooxygenase 2 activation (Rong and others 2016). Transdifferentiation may, in addition, utilize NOTCH pathway signaling (Cui and others 2018). Interestingly, glioma cell transdifferentiation appears not to be limited to endothelial cells since there is evidence that tumor cells can differentiate into pericytes, which express PDGFRβ and release VEGF and Ang-1 to stabilize newly formed blood vessels. This occurs as the endothelial cells themselves recruit glioma stem cells, which are then induced to become pericytes predominantly by TGF-β (Cheng and others 2013).

In the setting of HGG, endothelial cells and pericytes have additional roles beyond the formation and maintenance of the vasculature itself, as both cell types are critical in the maintenance of the perivascular niche. The concept of a niche in glioma biology is of a particular type of microenvironment within the tumor, such as perivascular, perinecrotic, or invasive, in which conditions favor a high degree of tumor stemness and glioma stem cells are accordingly most numerous (Schiffer and others 2018). Since glioma stem cells are the predominant driver of tumor growth and expansion, niches have a particular biological and therapeutic significance. In the perivascular niche, many of the factors that drive glioma stemness and invasion are secreted by endothelial cells and pericytes (Abdel Hadi and others 2018; Li and others 2019); furthermore, pericytes have been suggested to contribute toward other growth-promoting aspects of the perivascular tumor microenvironment, including immunosuppression and the maintenance of a hypoxic state at the margin of the tumor, which occurs in part by the formation of hypercontractile pericyte–glioma cell fusion hybrids that are associated with abnormally constricted blood vessels toward the tumor edge (Caspani and others 2014).

Alongside the various roles that endothelial cells play in supporting HGG growth, they have an intriguing role as tumor suppressors in the early stages of tumorigenesis via ephrin-B2 signaling, which compartmentalizes immortalized cells and inhibits invasion (Krusche and others 2016). However, fully transformed glioma stem cells appear able to bypass this mechanism by up-regulating ephrin-B2 ligand expression, which recruits a different signaling pathway to promote migration through evasion of ephrin-B2–mediated endothelial repulsion and an increase in repulsion between tumor cells themselves (Krusche and others 2016).

## Therapeutic Implications of Host-Glioma Interactions

The standard treatment plan for HGG usually includes an initial surgical resection, followed by radiotherapy and, for some patients, chemotherapy (Tan and others 2020). Despite the multimodal approach to treatment, however, recurrence rates are high, and the 5-year survival for glioblastoma, the most common form of HGG, is still <10% (Schaff and Mellinghoff 2023). A major reason for this poor outlook is the diffusely infiltrative nature of gliomas (Sahm and others 2012), which leads to difficulties in completely resecting tumors and delivering radiotherapy in a way that spares unaffected tissue from irradiation. In addition, many of the specific interactions with host cells described here that facilitate HGG growth and invasion contribute toward treatment resistance. For example, TMs that couple tumor cells via gap junctions enable a degree of resistance to radiotherapy, given that the integrated TM-connected network allows the buffering of damage-induced increases in Ca^2+^ levels in individual cells (Osswald and others 2015). TM networks also drive an exuberant tumor growth response following surgery as well as resistance to chemotherapy (Weil and others 2017). Not only are the standard interventions often ineffective, in some cases they can actually promote tumor growth and recurrence. A good example of this is the recently documented ability of temozolomide, the standard first-line chemotherapy for HGG, to drive glioma cell transdifferentiation and neovascularization (Baisiwala and others 2019). There is therefore a major unmet need for new therapeutic approaches to HGG. Many of the recent breakthroughs in understanding how HGGs interact with surrounding host tissues to facilitate growth and invasion could provide conceptually novel treatment targets with the potential, in theory at least, to drastically improve patient outcomes.

The critical role of neuronal activity in HGG progression is one such recently established phenomenon that readily suggests a variety of targets that could be exploited for therapeutic gain. Venkatesh and others (2019) demonstrated that activity at the functional glutamatergic synapses formed between host neurons and HGG cells drives tumor growth; this led the authors to examine the effects of perampanel, a pharmacologic blocker of AMPA receptors, which mediate basal neurotransmission at these synapses. As expected, this had a strong inhibitory effect on the growth of xenografts of pediatric HGG in mice. This is an exciting observation as glutamate receptors in general are an eminently druggable target and numerous antagonists to specific subtypes exist. Moreover, the full synaptic integration of HGG cells into functional circuits suggests potential therapeutic targets beyond the synapses themselves: activity-regulated secreted growth factors, ion channel function, and gap junction coupling could all potentially be targeted for therapeutic gain. Some of these approaches have now been attempted in animal models. The activity-dependent secretion of NLGN3, an important driver of HGG growth, was shown to be dependent on an ADAM10-mediated cleavage event. Accordingly, the ADAM10 inhibitor GI254023X robustly blocked the growth of HGG xenografts in mice (Venkatesh and others 2017). In later experiments, carbenoxolone or meclofenamate, blockers of the gap junction–mediated amplification of neuronal activity–dependent K^+^ currents, proved similarly effective in the same mouse models (Venkatesh and others 2019). Aside from glutamatergic synapses, glutamate release via the xc^–^ exchanger is important for various aspects of HGG growth and invasion, and elevated xc^–^ expression is an independent predictor of poor survival in patients with HGG. Sulfasalazine, an xc^–^ inhibitor, was shown to inhibit tumoral glutamate release in 9 human patients with HGG (Robert and others 2015), although results from small-scale clinical trials of sulfasalazine have been disappointing (Robe and others 2009). The drug seems to reduce peritumoral edema but have little direct cytotoxic effect on tumor cells (Sehm and others 2016), and it may be that its greatest potential lies as an adjunct to a conventional chemotherapy.

HGG growth depends critically on the tumor’s ability to interact with and regulate the responses of immune cells in the surrounding microenvironment, making these interactions very attractive candidates for new treatment approaches. Several immune cell–targeting interventions have been attempted in animal models, most commonly aimed at the critical TAM population. These have been subjected to reduction/depletion (Brandenburg and others 2016) and repolarization toward a less protumorigenic expression profile (Pyonteck and others 2013), as well as various measures to reduce recruitment, including blockade of the CX3CL1/CX3CR1 system, periostin, or osteopontin (Wang and others 2022). All of these interventions have produced therapeutic benefit in animal models and/or patients (Andersen and others 2021). As the importance of immune cells in the glioma context has been established for longer than that of neuronal activity, immune-modulating approaches to HGG therapy are correspondingly much more advanced, and there are currently ongoing clinical trials for a number of pharmacologic agents. These include multiple inhibitors of the CSF-1 receptor, such as emactuzumab, pexidartinib, cabiralizumab, and BLZ845, which act to prevent recruitment of TAMs and to repolarize their transcriptional profile away from a protumorigenic state, as well as inhibitors of the transcription factor STAT3, such as WP1066, which act downstream of CSF-1 receptor activation to regulate the expression of a number of cytokines and repolarize TAMs. Plerixafor, an antagonist of CXCR4, the receptor for the key TAM recruitment factor CXCL12, represents the third main class of compounds currently at the clinical trial stage (Buonfiglioli and Hambardzumyan 2021; Wang and others 2022). There may be a further application of inhibitors of TAM recruitment to HGG as an adjunct to oncolytic virotherapy, another new approach that employs engineered viruses to cause direct destruction of tumor cells and the promotion of an antitumor immune response, which confers lasting efficacy. By contrast, the early host innate immune response hinders the efficacy of the therapy as it targets the viruses directly, preventing replication and spread. Therefore, a transient block of TAM recruitment has the potential to enhance the efficacy of viral treatment, and this has indeed been shown to be the case in a mouse model of HGG (Han and others 2015).

The PD-1/PD-L1 signaling axis is another major target for immunotherapy in a variety of cancers, as it plays a vital role in inhibiting immune responses and promoting self-tolerance thorough modulating the activity of T cells, activating apoptosis of antigen-specific T cells, and inhibiting apoptosis of T_regs_. Gliomas evade the T-cell response partly through their expression of PD-L1, and this signaling axis has proved an attractive target for the development of novel therapeutics; some of these have already entered clinical trials in human patients (Shu and Li 2020). CheckMate 143 was the first such substantial clinical trial, examining the effect of the PD-1–targeting monoclonal antibody nivolumab. However, despite strong underlying preclinical evidence, nivolumab did not significantly improve survival in a cohort of patients with recurrent glioblastoma (Filley and others 2017). The reasons for this failure are not entirely clear but could include the fact that HGG expresses multiple mechanisms for T-cell inhibition so that inhibition of one may be circumvented by the ongoing activity of others. A parallel strand of work has focused on investigating the use of nivolumab and other PD-1–targeting drugs in combination with treatments such as temozolomide and radiotherapy. This approach has met with some success in animal models (Kim and others 2017; Wainwright and others 2014).

On the other hand, T cells can be specifically activated against various tumor types, including HGG, by chimeric antigen receptor (CAR)–T-cell therapy. CAR–T-cell therapy involves the generation of T-cell receptors specific for key tumor antigens that are then expressed in host T cells in vitro. The subsequent reintroduction of these T cells into a patient stimulates an antitumor response. This has been remarkably effective in several solid tumors, in particular melanoma, and has recently been tried in glioblastoma in two small clinical trials. By using T-cell receptors engineered to recognize tumor-specific variants of the EGFR receptor (amplification of which is a signature of IDH–wild type glioblastoma) or a tumor-specific interleukin receptor antigen, rapid and dramatic responses were achieved (Bagley and others 2024; Choi and others 2024). Unfortunately, in one of the trials, this good response was transient in most patients (Choi and others 2024). Nonetheless, these results establish that this approach can lead to effective tumoricidal activity.

Similar to PD-1/PD-L1 inhibitors and CAR–T-cell therapy, antiangiogenic approaches constitute a further therapeutic modality that has been repurposed to treat HGG following a significant record of successful use in the context of other malignancies. The anti-VEGF monoclonal antibody bevacizumab is in current routine clinical use for a variety of advanced carcinomas, including colorectal, renal, and gynecologic (Garcia and others 2020). The extensive tumor vasculature seen in HGG and the importance of the interactions between tumor cells and endothelial cells (as well as other vascular elements) for tumor growth provide a rationale for the use of this approach. Despite this, bevacizumab used as a single agent has not been shown to extend survival in patients with glioblastoma, despite sometimes impressive initial symptomatic and radiologic responses (Melhem and others 2023). Again, this could be a result of the redundancy of the targeted mechanism in that HGGs possess a suite of mechanisms for neovascularization, including not only those that are VEGF dependent but several that are not, such as vascular co-option and vascular mimicry. Interestingly, the lack of efficacy of bevacizumab may relate to the involvement of TAMs, with anti-VEGF therapy leading to the accumulation of these cells, which in turn was associated with poorer survival in patients with recurrent glioblastoma (Lu-Emerson and others 2013). As with other approaches that have proved ineffective when used alone, VEGF inhibitors are now being considered in combination with therapies such as temozolomide (Chinot and others 2014) and PDGFR-β inhibitors (Liu and others 2018). The development of bispecific antibodies as a single therapeutic agent with multiple targets represents a variation of simple single-agent combination therapy. Such antibodies have been developed to be directed against VEGF and Ang-2, a molecule that promotes invasiveness and angiogenesis, and these have proved effective at reprogramming the tumor microenvironment and extending survival in mouse xenograft models (Kloepper and others 2016).

While the treatments described thus far have been devised to target specific molecular mechanisms, a wholly different approach has been taken in the development of tumor-treating fields. These are alternating electric fields delivered to the patient’s scalp via wearable electrodes, and at the right intensity and frequency, these fields are able to exert physical forces on charged particles known as *dipoles*. This gives them the potential to modulate a variety of mechanisms, including host-glioma interactions, that promote or support tumor growth (Rominiyi and others 2021). Among these is the recruitment of immune cells to the tumor environment, which it achieves by disruption of the nuclear envelope, cytosolic DNA release, and consequent activation of the STING and AIM2 inflammasomes (D Chen and others 2022). This could directly counteract the immune-dampening mechanisms deployed by HGG cells described here.

## Conclusions

Bidirectional communication between tumor cells and the surrounding host tissue is critical to the progression of HGG, playing an active role in the reprogramming and modulation of the behavior of surrounding cells in order to reshape the tumor microenvironment in ways more conducive to tumor growth. A myriad of molecular pathways and processes mediate these interactions, for example paracrine signaling, synaptic transmission, and gap junction intercellular communication, and these are a rich source of potential therapeutic targets for the novel treatments for HGG that are so critically required. While understanding of host-glioma interactions has increased exponentially in just the last few years, much work remains before these mechanisms are fully elucidated. Nonetheless, development and testing of new therapies aimed at blocking or modulating host-glioma interactions are already well underway. Several of these have shown promise in preclinical disease models and are candidates for entry into clinical trials. A few agents have already reached the clinical trial stage, although by and large results in this setting have thus far been slightly disappointing. In some cases, this may represent a failure to optimize the dose or delivery of the agent, which can be addressed in future studies. In other cases, however, it may be that the agent works best in synergy with an orthogonal approach to target multiple mechanisms supporting tumor growth, and a number of therapies that have not shown a significant treatment effect when used alone are currently being taken forward on this basis.
